# Designing a Flexible and Inclusive Approach for Public and Community Involvement in Research With People Who Are Homeless or Vulnerably Housed: Critical Reflections From the *I Am More Than…* Project

**DOI:** 10.1111/hex.70325

**Published:** 2025-06-13

**Authors:** Mel Hughes, Kate Jupp, Cathy Beresford, Jim Robertson, Annabel Wathen, Tanya Bailey, Siobhan Lennon‐Patience, Mike Graham, Deano Pickering, Helena Posnett

**Affiliations:** ^1^ Faculty of Health and Social Sciences Bournemouth University Bournemouth UK; ^2^ Bournemouth University Bournemouth UK; ^3^ The Lantern Trust Weymouth UK; ^4^ The HealthBus Trust Bournemouth UK; ^5^ Help and Kindness Dorchester UK; ^6^ Dorset Healthcare NHS Foundation Trust Poole UK

**Keywords:** community, co‐production, homelessness, inclusive research, participatory, partnerships, public involvement

## Abstract

**Background:**

People who are homeless or vulnerably housed face significant health inequities and yet are rarely involved in health and social care research as participants, public contributors or co‐researchers. The *I Am More Than…* project was developed to address this lack of inclusion by working in partnership with community organisations and individuals with lived experiences of being homeless or vulnerably housed.

**Objective:**

To co‐design a flexible and inclusive approach for public and community involvement in research (sometimes referred to as public and patient involvement or PPI).

**Methods:**

The project drew on a range of co‐production and participatory research strategies, with input from various stakeholders, to identify and address enablers and barriers to participation. Community researchers with lived experience of homelessness or being vulnerably housed were central to all stages of the project, including the co‐design of strategies to engage people through conversations, interviews, creative outputs and stakeholder events.

**Findings:**

We identified core principles for inclusive public and community involvement in research. including the need to: develop relationships first and tasks second; gain a deep understanding of communities and their priorities; harness lived experience expertise; however someone may choose to share it; go at the pace of trust; and work in partnership with voluntary and community sector organisations who are embedded in their communities.

**Conclusion:**

Developing a flexible and inclusive approach to public and community involvement in research required shifting from a transactional approach to a relational approach. The project demonstrated the importance of building trust, working in partnership and being flexible and responsive to people's everyday realities for genuine inclusion to be achieved.

**Patient or Public Contribution:**

This is a co‐produced project between a university, an integrated care system, two community organisations and people experiencing homelessness or being vulnerably housed. Individuals with lived experience participated as community researchers, shaping the design, methods and outcomes. Their contributions are detailed throughout the paper, which is co‐authored by members of the project team, including staff from two community organisations and one community researcher.

## 1 Introduction

This paper shares critical reflections, insights and lessons learned from the *I Am More Than…* project; a collaboration between people with lived and living experience of being homeless or vulnerably housed, volunteers and staff who support them; and health and social care providers and researchers, to co‐design a flexible and inclusive approach to research participation and public and community involvement (sometimes known as public and patient involvement or PPI). The project is a response to the inclusive research agenda and the need for communities underserved by research to lead in co‐designing how they want to be involved if more diverse participation is to be achieved. The title *I Am More Than…* came from community researchers (with lived experience of the topic being explored) who identified the starting point for having a voice in research as being seen as more than homeless.

## 2 Aim

The aims of the *I Am More Than…* project:

1. To create inclusive, shared spaces for people with experience of being homeless or vulnerably housed, to share with and educate the health and social care research workforce on what having a voice and being involved in research means to them.
2. To understand the barriers and enablers to research participation and involvement from a homeless perspective.
3. To co‐create inclusive opportunities for people who are homeless or vulnerably housed to have a voice in shaping health and social care research.

This paper focuses on the iterative process of developing flexible and inclusive ways of working to engage people with experience of being homeless or vulnerably housed as community researchers and participants to achieve Aim 1 to create inclusive shared spaces, and what this entailed.

The paper is written from the perspectives of the community partners and a community researcher, with some co‐authors' voices kept distinct to highlight these specific perspectives.

The project is part of the Research Engagement Network (REN) Programme, an initiative by NHS England providing funding to integrated care systems (ICSs) across England to bring the voice of people and communities into health and care research [1, 2], with additional funding to embed the approach from the NIHR's Clinical Research Network Wessex. *I Am More Than…* started in October 2023 and is now in its third phase, with funding for Phase 4 starting in June 2025. Findings from Phases 2 and 3 (aligned to Aims 2 and 3, respectively) are explored in separate papers (pending publication) and include examples such as 18 changes to the local ICB's working with communities vision document, reflecting a shift from a listening approach to more active collaboration with marginalised communities. Phase 4 will enable us to evidence how the implementation of this way of working across multiple health research projects is effective in increasing the diversity of public and community involvement in research (in this case, specifically people who are homeless and vulnerably housed) and in addressing health inequalities through the research design they inform.

Through all phases of the project, specialist community partners The Lantern Trust and The HealthBus Trust worked with Bournemouth University to recruit, mentor and support 11 community researchers with lived experience of homelessness or being vulnerably housed, to undertake conversations within their communities and to share their findings with research and health and social care providers leading to the involvement of more than 125 people. Community researchers were offered the flexibility to be involved without specific expectations or requirements for regular or ongoing commitment. This study formed the basis of an approach to harnessing the lived experience expertise of people with experience of being homeless or vulnerably housed in health and social care research across Dorset, a largely rural and coastal county, in the south of England, recognised for high levels of homelessness [3, 4].

## 3 Background

From the start, we acknowledged the significant health and social inequities faced by people who are homeless or vulnerably housed and their frequent absence from research participation. The *I Am More Than…* project, sought to actively involve people in research to provide a deeper understanding of the challenges and ensure that strategies to increase diverse participation were co‐designed. Beresford [5], highlighted that if diversity and the barriers to diversity are not addressed, ‘participation is likely to be partial, and reflect broader social divisions and exclusions’. Not seeking lived‐experience expertise or involving people in research risks reinforcing rather than addressing health and social inequities. Approaches to public involvement in research (or PPI) are typically designed by those within the research community, seeking to invite people to become involved. The *I Am More Than…* project sought to flip the power by starting with homeless communities to design how they want to be involved and then creating opportunities to educate and build capacity within the research workforce to make this happen. From the outset, our approach was for this to be community‐led, with the university partners providing support and guidance rather than setting the agenda.

### 3.1 Homelessness in England and Dorset

Homelessness is an increasing concern. In England, between 2023 and 2024, there was a 10% increase in households requiring an initial assessment for homelessness assistance. A total of 358,370 households were assessed, with 324,990 determined to be homeless [6]. Rough sleeping is rising, and in June 2024, an estimated 8309 people were sleeping rough in England [7]. In Dorset, work is underway across both local authorities to address the issue, for example, the Homelessness and Rough Sleeping Strategy 2021–6 [8]. Bournemouth, Christchurch and Poole (BCP) in Dorset is one of the locations selected by the Royal Foundation—Homewards to develop local actions towards the trajectory of ending homelessness.

### 3.2 Health Inequities

Significant health inequalities affect individuals experiencing homelessness, including higher rates of illness and drastically reduced life expectancy. For people experiencing long‐term rough sleeping, life expectancy is 46 years for men and 42 years for women [9]. Pathway [10] highlighted the extent of health service exclusion associated with homelessness due to inflexibility, discrimination and stigma within the system. This exclusion contributes to morbidity and mortality rates [11]. Taylor et al. [12] reported the deaths of 1474 individuals experiencing homelessness in the United Kingdom in 2023, illustrating the urgent need for radical, collaborative action to address health and social care inequalities. One‐third of deaths among individuals experiencing homelessness are due to treatable conditions, including respiratory, gastrointestinal or cardiovascular diseases, or cancer, which could be improved with appropriate care [8, 13, 14].

A key focus from the outset of this project, therefore, was to increase the opportunities for people to share their lived experience expertise to improve the relevance, quality and impact of health and social care research to reduce these health inequalities.

### 3.3 The Need for System Change

Emphasis is increasingly placed on diversity in research participation and public involvement, which is crucial to high‐quality, relevant health research that does not reinforce healthcare inequalities [15, 16]. Darko [15] advocates for effective partnerships to meet the needs of different groups and overcome structural and cultural barriers to research involvement. Reports from initiatives such as the NIHR's Research Ready Communities Programme [17] and NHS England's REN development funding consistently show that communities are keen to be involved in research on their terms, but that infrastructure and processes need to change for this to be more accessible [1]. The *I Am More Than…* project reflects a flipping the power approach, shifting from training people with lived experience and communities to be involved in research to communities training the research workforce on how to be more inclusive. We were able to do this by dedicating time and resources to building on existing relationships between the university and the two community partner organisations developed through previous project work. This created the foundation for co‐designing the project from the outset.

### 3.4 Homeless Participation and Co‐Production in Research

Several researchers have specifically sought participation from people experiencing homelessness. For example, Hudson et al. [18] explored the perspectives of the homeless community about palliative care. The Hearth Study [19] focused on delivering primary health care to people experiencing homelessness, and members of the patient and public involvement group with lived experience were trained and supported to undertake peer interviews. The crisis report (Mackie 2014) also involved people with lived experience.

The extent to which people with lived experience have participated as co‐researchers, however, is not always clear. More recent studies [19, 20] have explicitly sought to include people with lived experience in advisory roles and as co‐researchers, advocating for more inclusive approaches. A recent report exploring the healthcare of people experiencing homelessness with diabetes [21] exemplified the value of meaningfully involving people with relevant lived experience. The report identified that Experts by Experience were crucial in shaping the project and focusing on the issues important to the community, including the need for empathetic and skilful care delivery that avoids causing disengagement. The Experts by Experience advocated for individuals with diabetes and experience of homelessness to be interviewed for the project, which had not been the original plan. The subsequent insights were reported as invaluable, and two interviewees later joined the project steering group.

During our review of the literature, examples of homeless individuals participating in research were limited. Opportunities to engage in broader health and social care research or contribute to research design appear to be sparse, particularly outside studies specifically focused on homelessness. A project with young people experiencing homelessness identified specific barriers to research involvement, including travel costs, time constraints, scepticism about the benefits and motivations of research, and digital poverty [22]. The project emphasised building confidence and trust, ensuring personal agency regarding involvement and providing a financial incentive. Participation and involvement needed to be on the individual's terms, with researchers adopting more inclusive approaches. Community‐led research is not without its challenges. Salway et al. [23] demonstrated that involving people with lived experience as community researchers can address health inequalities by building trust and relevance. However, they also highlighted the complexities, including ethical considerations and the need for appropriately resourced and skilled support to ensure meaningful participation. The *I Am More Than…* project sought to enable involvement in health and social care research with people experiencing homelessness or being vulnerably housed, by co‐designing together what form this should take and by drawing on the expertise of voluntary and community organisations to provide ongoing support and care.

## 4 The Approach

Table 1 explains the *I Am More Than…* approach developed through this iterative co‐design process. These principles evolved throughout the project from the underpinning methodology, which recognises the distinction between system world and lifeworld priorities and motivators [24]. In the system world, research is often seen as a clearly defined process: objective, and transactional, with participants providing data for researchers, frequently resulting in an unequal power dynamic [25]. In the lifeworld, people collaborating on projects have their motivations and desired outcomes, which need to be identified and acknowledged. Lifeworld priorities are typically based on values, relationships, communication and everyday realities. The project prioritises lifeworld principles by adopting the approach that people with lived experience and community partners determine priorities and direction, with academic researchers providing support, reversing traditional power dynamics [26]. At the start of Phase 2, and ahead of a second co‐delivered stakeholder event, all those involved in the project team (including the community partners) sought to identify what the emerging approach was and was not.

**Table 1:** *The I Am More Than…* approach to public and community involvement in research.

**Is…** A way of working. About relationships first, tasks second. Based on gaining a deep understanding of communities and their priorities. A personalised approach. Starting where people are. Harnessing lived experience expertise, however, someone may choose to share it, at the pace of trust. In partnership with the support and expertise of voluntary and community sector organisations embedded in their communities.	**Isn't…** A defined step‐by‐step process. Driven by schedules, time frames and deadlines. A formal role with a job description. Led by system priorities (we need four people who…). One size fits all. About training people with lived experience to fit into a system's way of working (although involvement may lead to other opportunities of their choosing).

These principles were identified by the communities involved as essential to an approach that is community rather than system‐led. A participatory approach is at the heart of the *I Am More Than…* project, but differs in that it is an inclusive and participatory approach to public and community involvement (often referred to as PPI). In 1969, Arnstein developed the pioneering Ladder of Participation, which demonstrated how citizen participation leads to citizen power in the decisions affecting their lives [27]. However, Arnstein herself acknowledged the simplicity of her approach, which implied a linear and hierarchical participation process. The reality can differ, particularly when working in participatory ways with individuals who are frequently excluded and disenfranchised, such as those experiencing homelessness.

Since Arnstein, understandings have evolved to offer a less hierarchical view of participation, recognising that involvement varies depending on the situation, and full citizen control is not always the only or desired outcome [28, 29, 30]. The *I Am More Than…* project focused on creating opportunities for voices to be heard, valuing all contributions and ensuring they make an impact. Flexible, fluid pathways were developed within this project to meet individuals where they are, promoting greater diversity in participation while offering progression for those seeking deeper or more regular involvement. We found that any attempt at more structured involvement was either viewed as system‐led or top down and imposed, and not of direct benefit to the homeless people involved, or reduced the level of engagement due to the nature of people's lives and their need for more ad hoc involvement, especially at the start.

Participatory research involves an evolving, collaborative process where diverse voices question the subject of study and the methods used. A key element is learning together to drive action, which requires creating supportive, relational spaces to generate new knowledge, which is just as relevant to creating an approach to public and community involvement in research. Different perspectives come together, and critical reflection and consideration of the possibilities beyond current realities are encouraged [31]. As with the system and lifeworld concept [24], involvement requires the creation of shared spaces where all priorities can be acknowledged and addressed. In the *I Am More Than…* project, this approach was central to providing an environment where individuals could contribute to co‐produced knowledge and reflect on their experiences.

At the heart of the success of I *Am More Than…* is partnership working with community organisations, building on the existing trusted relationships between them and the community they serve. The methodology acknowledges the ‘messiness’ of participatory approaches and community involvement, particularly with groups that face exclusion. Working in partnership means that rather than shying away from challenges and complexities, we have worked together, drawing on each other's expertise and skills to acknowledge and embrace them. Responsiveness and inclusivity have required flexibility and adapting to the needs of the individuals, for example, by creating fluid opportunities where people can dip in and out of a role depending on personal circumstances. The role of each community organisation's outreach or project workers has been invaluable. Being embedded in the organisation, a place of trust and repeated points of support for the everyday life challenges faced by people who are homeless or vulnerably housed, has meant that chance conversations, contributions in parallel, and being able to pick up on passions, ideas and concerns can happen as they surface, supporting spontaneity and reducing barriers and formality of having to attend set meetings. The project aimed to ensure that everyone who wanted to contribute could do so in a way that was right for them. Keeping the door open is essential for shifting power and moving beyond more traditional, formal approaches to involvement [32] and did lead to four community researchers becoming more regularly involved in meetings, workshops, stakeholder events and co‐authoring papers and outputs, as and when they chose to.

Collaborating with community partners and people with lived experience in the project requires resources for community organisations and the infrastructure to be in place to support sustainability. Part of our learning has been to shift attention from involving a single individual as a public contributor to research projects—a practice that can bring significant risk to the individual in terms of emotional labour and burden of involvement, to one that seeks to engage a diverse range of voices from a wider community, with the support of the voluntary and community sector organisation—an approach we concluded was more likely to address health and social inequalities, reduce the emotional labour of involvement and have greater impact.

## 5 The Feel

In developing an inclusive approach to diverse public and community involvement based on lifeworld motivators, we learned to prioritise experiences of and feelings about involvement [33]. Central to our philosophy is the ‘*feel’* of the project, grounded in the shared principles and values of relationship first, task second and going at the pace of trust. This relational approach has been embedded at all levels and stages of the project—it is not simply ‘rolled out’ when in contact with people experiencing homelessness. It is an approach that is role‐modelled throughout, from project steering group meetings, to conversations on the pavement outside a community drop‐in, and in all external communications, enabling others less familiar or more sceptical, to experience ‘the feel’ firsthand and in their familiar environment.

This lifeworld approach prioritises positive and validating experiences for people, avoiding the transactional or extractive models that we found often leave individuals feeling exploited and questioning the value of their involvement in future research, compounding existing barriers to participation. While inclusive involvement can sometimes be challenging and uncomfortable [34], the project team worked hard to co‐create an atmosphere where everyone involved felt valued and heard. This centres on ensuring agency to contribute, with all individuals truly listening to one another and co‐designing how best to act, leading to change [35].

## 6 Methods

Articulating the *I Am More Than…* approach to public and community involvement is complex, and Table 1 illustrates the principles. As an iterative process to co‐designing inclusive ways of working, we created reflexive spaces for all project members to contribute to its evolving design, including meetings, workshops and one‐to‐one support. Throughout this process, we continually assessed our adherence to the core values of an inclusive participatory approach, enabling us to identify key facilitative features and recognise when system challenges were obstructing them.

Ethical approval was obtained from the University's Research Ethics Committee to undertake and publish findings from the project and was conducted according to the University's Research Ethics Code of Practice.

## 7 What This Way of Working Involved

1. Collaborating with people who are homeless or vulnerably housed, along with volunteers and staff who support them, we developed an approach based on lifeworld motivators and priorities. The core project team (BU PIER, Lantern Trust and HealthBus Trust, overseen by a broader project steering group) recruited, mentored and supported community researchers with lived experience of homelessness or being vulnerably housed to join or take a step back at any time.
2. We developed a three‐stage cyclical process where the university team supported and mentored staff from the partner community organisations, who in turn supported and mentored community researchers. Each stage informed the next with opportunities to learn from each other.
3. We shifted from a transactional approach to research involvement to a relational approach, focusing on building relationships, listening deeply, understanding the enablers and barriers from the perspectives of individuals and community organisations, and going at the pace of trust. We ensured that words led to action by bringing people who are homeless or vulnerably housed, volunteers and staff, together with members of the research workforce, to design the next steps [1]. Primarily, individuals and community partners were involved on their terms. Community leads were identified from each partner organisation (the HealthBus Trust and the Lantern Trust) to be part of the core project team. They supported community researchers in whatever ways they wanted. This involved navigating the everyday challenges the community researchers faced. What this looked like in practice ranged from community researchers engaging people from their wider community in conversations, collecting stories, completing ‘I am more than’ tiles, making films and engaging stakeholders through shared lunches and community events, with more specific examples shared later in this paper in the ‘Community Reflections’ section.
4. Finally, we ensured support and resources were available and responsive at each stage. The community organisations were not exclusively gatekeepers or bridge builders; they were active partners in undertaking and supporting the activity and part of the learning process. As the community reflections illustrate in the ‘Findings’, the support and resources needed varied between community organisations, reflecting differences in their structure, capacity, research experience, priorities and the uniqueness of the communities they serve. The university team provided structured and informal training, mentoring and support to the community organisations, enabling them to lead on community researcher involvement. Support was initially frontloaded, with the university team involved in all activities. This then transitioned to weekly meetings with ad hoc individual support as required. Over time, the university's role shifted to guiding how to embed the approach, build further capacity across voluntary and community sector organisations and share the findings more widely, such as supporting the co‐authorship of this and other papers and outputs.

### 7.1 Through Ongoing Co‐Design, Our Flexible and Inclusive Approach to Public and Community Involvement Included

- Role modelling a creative, flexible and sensitive approach to research involvement and collaboration for all involved, for example, by starting each meeting or encounter with a focus on well‐being and needs before focusing on tasks.
- Nurturing sometimes fragile relationships with community researchers through flexibility and support. For example, ensuring that their involvement is sensitive to other events in their lives.
- Supporting community researchers to draw on their connections, strengths and skills to engage with other people experiencing being homeless or vulnerably housed. With arms‐length support and access to resources such as art materials and equipment, community researchers chose and developed creative approaches that fitted with their individual strengths and preferences to design practical and ethical ways of engaging with individuals whose voices are seldom heard. This included informal approaches to conversation, meeting people in trusted community spaces and adapting methods as explained by Jim, Annabel and Tanya in their reflections below.
- Facilitating impromptu and opportune conversations and involvement throughout the week, for example, when sitting on the steps outside a drop‐in or when driving to an appointment.
- Connecting on a human level through shared lived experiences, highlighting the importance of listening and valuing every individual's contribution and voice.
- Responding to the emotional labour of being a community researcher, such as being challenged by a participant as to whether they had ‘changed sides’.
- Providing mentoring and support for community organisations through weekly online meetings, email and telephone contact, as well as co‐delivering events and workshops.
- Creating opportunities, such as stakeholder events and workshops [1, 36], to bring together people from the homeless community and the research community to learn from each other and ensure that learning impacts practice.
- Ensuring that academic researchers are open to inclusive ways of working by bringing them together with community researchers and organisations.

The Lantern Trust and the HealthBus Trust engaged with the *I Am More Than…* project very differently from each other reflecting the uniqueness of the services, each bringing distinctive strengths, and increasing the diversity and reach of the project. Individuals accessing the HealthBus Trust may be unwell, so the *I Am More Than…* project fits around them, engaging only when appropriate. In contrast, the Lantern Trust held structured local events due to the availability of dedicated rooms and the more regular way people engage with their services.

Initially, both community partners were unsure about how it would work practically to achieve the project's aims. Understanding the details of what the community needed was crucial, and time was spent visiting, envisaging together what it could and equally importantly, couldn't be through their eyes. Community partners identified what was needed, including resources for refreshments, gift cards and art canvases to collect people's initial perspectives. The art canvases (tiles) became the hook by which many participants and community researchers started a conversation by seeking to capture *I Am More Than*…

Community partners from both organisations have reflected on their involvement in the project to date.

## 8 Findings

In the *I Am More Than…* approach, we found that the community organisations were best placed to know what, where, who and how community research could work with the people supported by their services. How this worked in practice depended on the needs of community organisations, the workers and the community researchers, which changed over time as confidence and trust grew or as new people joined the project team.

We learned that taking time to unpick and understand how people and community partners want to be involved can accelerate the development of trust and understanding of the community's priorities and concerns. Individuals and community partners must not be left feeling they have been used and discarded. Partnering with community organisations has been fundamental to the success of the project. We found that only with established, trusted relationships will a community organisation risk their relationship with the community they serve by being involved in research. Involvement in research is not risk‐free; it must be explored together to provide the foundations of building trusting and lasting partnerships, working with benefits for all involved.

We found that the *I Am More Than…* approach is centred around its flexibility, the strength of the community partnerships and the openness of the system world partners to change processes and practices to facilitate more inclusive and flexible forms of public and community involvement. This is explained in more detail in the ‘Community Reflections’.

## 9 The Approach in Action—Community Reflections

### 9.1 Community Reflection 1

**Table:** 

Jim Robertson *Community Researcher with lived experience of homelessness* Drugs and homelessness are connected for me, it's unlikely I'd have become homeless without drugs. I wanted to get involved because I have commercial experience in project management. The *I Am More Than* project sounded like an interesting challenge. It appealed to my sense of justice, especially around reducing the stigma of homelessness. I already wanted to change my career direction. This project offered a chance to explore the charitable sector and see if it suited me. We hosted events, provided food, and invited people to talk to us. As a community researcher, I interviewed people, asking them to share their stories of homelessness. For many, it was their first time speaking about their experiences. Some shared a few paragraphs, while others gave me pages and pages. In the second phase, I arranged access to a hostel, which worked well. I was able to engage residents directly, and word spread, leading more people to get involved. There are challenges when working with vulnerable people, so I completed Safeguarding Adults Training for Volunteers – Level 1, which was already available to staff and volunteers at the Lantern Trust to ensure I could carry out the conversations ethically and responsibly. The *I Am More Than* project helped me meet new people through my interviewing, which broadened my social group. Involvement led to other opportunities like volunteering and training I wouldn't have accessed otherwise. Because of my interest and participation, I was able to speak up about wanting to use my lived experience to work with drug users. It also built my relationship with Tanya at the Lantern Trust, who then understood what kind of work I was interested in and capable of doing. This project reminded me of the forgotten skills I had from my previous career. It gave me a sense of direction ‐ not just for work but in how I connect with people. Overall, it was a really good thing for me.

### 9.2 Community Reflection 2

**Table:** 

Annabel Wathen Operations, Training & Research Officer, the HealthBus Trust. For the last 3 years, I have worked for the HealthBus Trust, a GP‐led service providing accessible healthcare to individuals experiencing homelessness in Bournemouth. My role is non‐clinical, and my favourite part is driving the bus because I engage with people one‐on‐one through outreach work. This gives me the most opportunities for the *I Am More Than…* project, as I meet people, chat with them and build rapport. The HealthBus Trust was invited to be a community partner for the project because we engage with people who are homeless. However, it was important to look beyond that label to understand that individuals are more than their situation. The project title *I Am More Than…* reflects this sentiment: we are all more than the marginalising labels we might carry. I wanted to support individuals in expressing themselves beyond the title of homelessness because everyone has a story and multiple identities that define them beyond their current situation. When I got involved with the *I Am More Than…* project a year ago, I felt nervous about approaching people. This led me to consider how to build rapport before introducing the research, as I didn't want to make anyone uncomfortable. Project funding enabled me to purchase food and vouchers to recognise individuals' contributions. Their time is as important as mine, and it was vital for involvement to be worthwhile for them. We aren't asking for favours; we are acknowledging their value. I have found that simple actions like offering sausage rolls or a cup of tea help to break the ice and put people at ease. Sharing a meal can also reduce the pressure of formal research. I casually introduce the project by asking, ‘Will you help me with my project?’ This sparks curiosity and engagement. I invite people to write a sentence about themselves on a canvas. For instance, one young man, initially hesitant, shared that he is not just a 23‐year‐old living on the streets—he's a dad, a brother, and someone with career aspirations. These conversations often reveal sides of individuals they rarely discuss. Another time, I assisted a woman who couldn't read or write but wanted to be heard. I sat with her and transcribed her thoughts. It was a powerful moment of reflecting on her identity beyond her struggles. I have found that this project often marks the first time someone has considered their identity in depth, especially when their life has been dominated by survival. As well as building relationships and hearing individual stories, there are positive byproducts from the *I Am More Than…* project. People have spoken about the impact on their self‐worth and expressed that involvement has given them a sense of purpose. Some have even become quite emotional about it, feeling seen and valued. That has been a lovely takeaway. To involve people who are homeless in research, we have a responsibility to be part of the solution. It often comes down to the basics: empathy, communication, finding out about people, who they are and what they like. We have to build relationships and work with people to understand what is important to them.

### 9.3 Community Reflection 3

**Table:** 

Tanya Bailey Education, Training & Employment Coordinator, the Lantern Trust I work as the Education, Training, and Employment Officer with the Lantern Trust in Weymouth, which started as a safe space for people experiencing homelessness to get a cup of tea and have a chat if they were experiencing difficulties. Over time, it's grown into a hub where people can access vital services under one roof—housing and benefits advice, clinical support, and more. My role involves engaging clients from the Rough Sleeper's Initiative to help them access opportunities for education, training, and employment. Building relationships is key to guiding them onto this pathway successfully. Last year, my role expanded to include the *I Am More Than…* project, which aims to give people experiencing homelessness a real voice in research. The idea is not just to gather stories but to explore ways of actively involving those with experience of homelessness or being vulnerably housed in the research process. It's about breaking down the walls that typically separate researchers from the communities and people they're studying to ensure that those with lived experience have a say in research. However, it was difficult to get to grips with at first and getting people engaged has been tough. Many clients are understandably cautious, asking, ‘How is this going to help me?’ Research often feels distant, like something done to people, not with them, and they wonder if it will really make a difference. To address this, we tried to make the process inviting and low‐pressure, holding casual coffee meetups where people could just come, chat, and see what it's about. We also arranged drop‐in events where clients could sit down one‐on‐one to share their stories with one of our community researchers: four individuals with lived experience who we supported. The approach has brought out some powerful stories around housing struggles, family breakdowns, and the day‐to‐day challenges of homelessness. But it's not always easy to engage a range of people. Most individuals involved are men who access our service. I have tried to include women, with two coming to one of our events. Building trust takes time and it's essential to keep offering encouragement and one‐on‐one opportunities to build those relationships. We aim for a broad range of voices, but the barriers are real. Reflecting on the past year, I've seen how important flexibility and persistence are in making research inclusive. *The I Am More Than…* project isn't just about undertaking research—it's about giving people the opportunity to direct the process, reclaim their stories, and see themselves as more than their circumstances. However, it is not a straightforward process to navigate, and we had to learn how to make the project work, but it's an important step in valuing different voices and working inclusively.

## 10 Final Reflections and Insights

Undertaking the *I Am More Than…* project has been described by those involved as rewarding, humbling and challenging. The approach required all stakeholders to reflect critically on themselves, their view of homelessness and working with marginalised communities and to explore different ways of engaging with people. As recommended by the National Institute for Health and Care Research (NIHR) [37] and Co‐Production Collective [38], we have thought beyond traditional notions of what it means to be inclusive and collaborative. Flexibility, sitting with discomfort and thinking outside of the box have been critical. This can be particularly challenging for those of us who prefer clarity, clear guidance and processes offered by the system world. We have found that working with people who are marginalised or have complex lives requires a more adaptive approach, which acknowledges what is most important to them, grounded in their reality, rather than in the demands of the system world. The role of those of us facilitating the involvement is to be able to buffer the different needs and priorities of the system and the lifeworld. We have learned that it is okay to hold a meeting when one person turns up because cancelling would jeopardise the trust, devalue lived experience expertise and contradict the relationship‐first approach. We found that momentum builds, and more people get involved. It is about embracing challenges and collaboratively identifying pinch points to address uncertainty effectively, rather than deferring issues that may later become bigger barriers. Challenges are not failures but opportunities for growth and learning.

## 11 Conclusion

The *I Am More Than… inclusive* approach to public and community involvement in research demonstrates that for health and social care research to be inclusive and involve people experiencing homelessness or being vulnerably housed, it helps to work collaboratively with community partners, prioritising trust, relationships and flexibility. The challenges and barriers encountered in the *I Am More Than…* project were significantly outweighed by the benefits and opportunities it presented. Recognising difficulties and actively seeking collaborative solutions are crucial for success. A key learning point for us has been that there is no one‐size‐fits‐all approach; being open, flexible and responsive is essential. True inclusivity and responsiveness requires providing opportunities for community researchers to contribute on their terms as much or as little as they feel able to at any given time, ensuring their involvement is meaningful and accommodating to their circumstances. An inclusive approach to public and community involvement in research, based on lifeworld priorities and motivators, acknowledges difficulties, embraces the ‘messiness’ of the situation and creates a positive feel for people to come together to be involved in research.

## Author Contributions

**Mel Hughes:** conceptualisation, investigation, funding acquisition, writing – original draft, methodology, visualisation, writing – review and editing, formal analysis, project administration, data curation, supervision, resources. **Kate Jupp:** conceptualisation, investigation, funding acquisition, writing – original draft, methodology, visualisation, writing – review and editing, formal analysis, project administration, data curation, supervision, resources. **Cathy Beresford:** writing – original draft, writing – review and editing, visualisation, project administration, supervision, data curation. **Jim Robertson:** investigation, writing – original draft, conceptualisation, writing – review and editing. **Annabel Wathen:** conceptualisation, investigation, writing – original draft, writing – review and editing, visualisation, methodology, project administration, supervision, resources. **Tanya Bailey:** conceptualisation, investigation, writing – original draft, methodology, visualisation, writing – review and editing, project administration, supervision, resources. **Siobhan Lennon‐Patience:** funding acquisition, conceptualisation, writing – review and editing, visualisation, methodology, project administration, data curation, supervision, resources. **Mike Graham:** funding acquisition, writing – review and editing, conceptualisation, visualisation, supervision, resources. **Deano Pickering:** conceptualisation, funding acquisition, visualisation, writing – review and editing, supervision, resources. **Helena Posnett:** funding acquisition, writing – review and editing, conceptualisation, methodology, visualisation, project administration, supervision, resources.

## Conflicts of Interest

The authors declare no conflicts of interest.

## Data Availability

The data that support the findings of this study are available from the corresponding author upon reasonable request.
